# Synthesis and Characterization of Phosphinecarboxamide and Phosphinecarbothioamide, and Their Complexation with Palladium(II) Complex †

**DOI:** 10.3390/molecules27175564

**Published:** 2022-08-29

**Authors:** Masumi Itazaki, Kento Okabayashi, Takanari Matsutani, Tomoya Nochida, Toshiyuki Moriuchi, Hiroshi Nakazawa

**Affiliations:** 1 Department of Chemistry, Graduate School of Science, Osaka Metropolitan University, Sumiyoshi-ku, Osaka 558-8585, Japan; 2 Department of Chemistry, Graduate School of Science, Osaka City University, Sumiyoshi-ku, Osaka 558-8585, Japan

**Keywords:** hydrophosphination, phosphinecarboxamide, phosphinecarbothioamide, palladium(II) complex, hydrogen bond, *cis/trans* isomerization, crystal structure

## Abstract

Reactions of isocyanates/isothiocyanates with primary and secondary phosphines without solvent at room temperature afforded phosphinecarboxamide/phosphinecarbothioamide, respectively, in excellent yields. Furthermore, palladium complex Pd(COD)Cl_2_ was allowed to react with Ph_2_PC(O)NHPh (**1a**) to afford [Pd{Ph_2_PC(O)NHPh-κ*P*}_2_Cl_2_] (**3**). On the other hand, the reaction of Pd(COD)Cl_2_ with 1 eq. of Ph_2_PC(S)NHPh (**2a**) afforded [PdCl_2_{Ph_2_PC(S)NHPh-κ*P,S*}] (**4**). In the case of a 1:2 molar ratio, [PdCl{Ph_2_PC(S)NHPh-κ*P,S*}{Ph_2_PC(S)NHPh-κ*P*}]Cl (**5**) was formed. The newly obtained compounds were fully characterized using multielement NMR measurements and elemental analyses. In addition, the molecular structures of Ph_2_PC(O)NH(CH_2_)_2_Cl (**1j**), Ph_2_PC(S)NHPh(4-Cl) (**2c**), and **3**–**5** were determined using single-crystal X-ray diffraction.

## 1. Introduction

**Figure 1:**
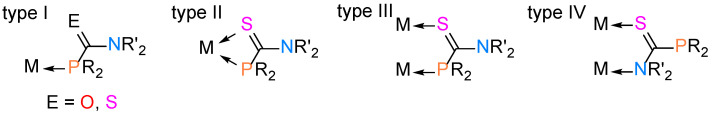
Typical coordination modes for phosphinecarboxamide and phosphinecarbothioamide.

Phosphinecarboxamides R_2_PC(O)NR′_2_ and phosphinecarbothioamides R_2_PC(S)NR′_2_ are phosphorus-analogs of urea and thiourea, and these are interesting compounds in coordination chemistry because of the variety of coordination modes. These compounds are known to act as a *P*-coordinated monodentate ligand (type I) [1,2,3,4,5,6,7], a *P,S*-coordinated bidentate ligand forming a four-membered chelate ring (type II for phosphinecarbothioamide) [8,9,10,11,12,13], and a bridging ligand for two metal centers (type III and type IV for phosphinecarbothioamide) [7,14] (Figure 1).

One of the most efficient methods for the synthesis of phosphinecarboxamide/phosphinecarbothioamide is the hydrophosphination of isocyanates/isothiocyanates. The reaction without a metal catalyst has some drawbacks (low yield and long reaction time) [15]. Therefore, metal-mediated methods have been developed, and there have been two reports on rare earth metals (La, Y, Eu, Er, Yb) [16,17], two reports on Th and U [18,19], one report on Fe [20] and one on Zn [21] for both isocyanates and isothiocyanates, and one report each on Zr [22], Cu [23] and Sb [24] for isocyanates to date. However, the removal of toxic metals after the end of the reaction is required when a metal compound is used. Therefore, the development of metal-free methods has been researched.

Recently, the transition-metal complex bearing 1,1′-bis(diphenylphosphinecarboxamidyl)ferrocene Fc(NHC(O)PPh_2_)_2_ as a ligand has been reported to exhibit unique properties. The tetranuclear Pt complex with six Fc(NHC(O)PPh_2_)_2_ has a hollow cage structure [25], and the macrocyclic dimer of Fc(NHC(O)PPh_2_-AuCl)_2_, induced by aurophilic interactions, shows helical chirality induction into the ferrocene core when using chiral proline methyl ester hydrochloride [26]. Although phosphinecarboxamide has attracted attention as a ligand, no examples have been reported comparing the ligand behavior of phosphinecarboxamide and its thiocarbonyl analog (phosphinecarbothioamides) with the same transition-metal complex precursor. In this study, we report the synthesis of isocyanate/isothiocyanate without using a transition-metal catalyst and the reactions of Pd(COD)Cl_2_ (COD = η^2^:η^2^-1,5-cyclooctadiene) being a typical Pd(II) complex with Ph_2_PC(E)NHPh (E = O, S). We found the first example of the thermal *cis*/*trans* isomerization of a transition-metal complex with phosphinecarboxamide and phosphinecarbothioamide having intra- and/or inter-molecular hydrogen bonds. A part of this work was preliminarily reported in [27].

## 2. Results and Discussion

### 2.1. Synthesis of Phosphinecarboxamide and Phosphinecarbothioamide

**Scheme 1:**
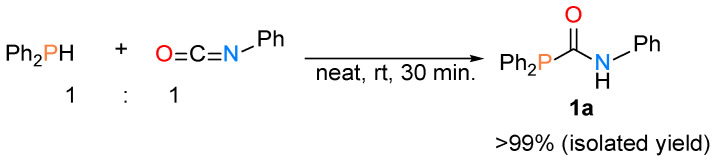
Hydrophosphination reaction of phenylisocyanate with diphenylphosphine.

Previously, we found that iron catalyzes the hydrophosphination of the unsaturated C–C bonds of alkynes and vinylphosphines with secondary phosphines [28,29]. In these reactions, phosphine compounds do not act as a catalyst poison. Therefore, we thought that the hydrophosphination reaction of isocyanates and isothiocyanates may be adaptable in our system. We performed the reaction-condition screening of the hydrophosphination of phenylisocyanates and found that no solvent was the key reaction condition. The desired hydrophosphination product, Ph_2_PC(O)NHPh **1a**, was obtained within 30 min in >99% yield at room temperature when catalyst and solvent were not used Scheme 1.

**Scheme 2:**
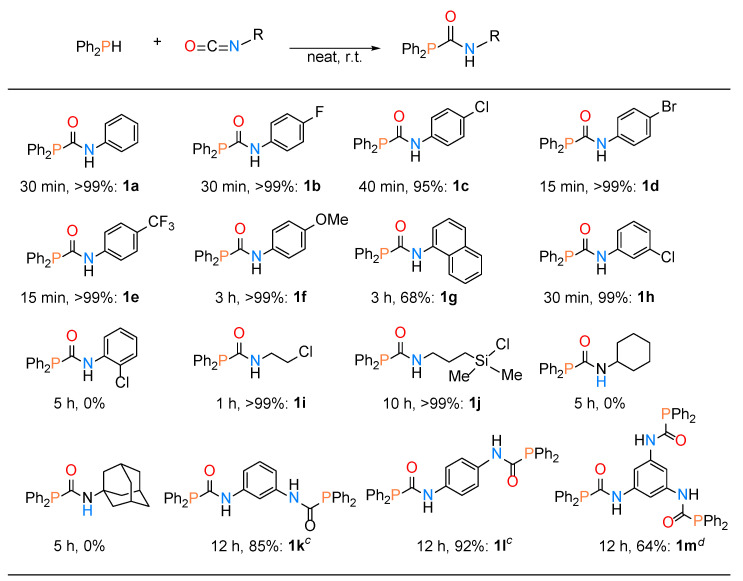
Scope and limitation for hydrophosphination of isocyanates with diphenylphosphine ^a,b^. ^a^ Ph_2_PH: isocyanate = 1:1 molar ratio. ^b^ Isolated yield. ^c^ 80 °C, 2 equiv. of Ph_2_PH was used (vs. isocyanate). ^d^ 80 °C, 3 equiv. of Ph_2_PH was used (vs. isocyanate).

We examined the substrate scope and limitation for the hydrophosphination of isocyanates with diphenylphosphine (Scheme 2). Phenylisocyanates with an electron-withdrawing group such as F, Cl, Br, and CF_3_ at the *para* position and Cl at the *meta* position on the phenyl ring were readily converted (within 40 min) into the corresponding phosphinecarboxamides in excellent yields (**1b**, **1c**, **1d**, **1e**, **1h**). The reaction of *para*-methoxyphenylisocyanate and that of 1-naphthylisocyanate took longer (**1f**, **1g**). Isocyanates with aliphatic substituent were also adaptable to this reaction (**1i** and **1j**). When 2-chlorophenylisocyanate, sterically bulky cyclohexylisocyanate, and 1-adamantylisocyanate were used, the desired products were not yielded. The double hydrophosphination of 1,3- and 1,4-diisocyanatobenzene were found to yield the desired products, **1k** and **1l**, in 85% and 92% yields, respectively. In addition, the triple hydrophosphination product **1m** could be obtained in 64% yield.

**Scheme 3:**
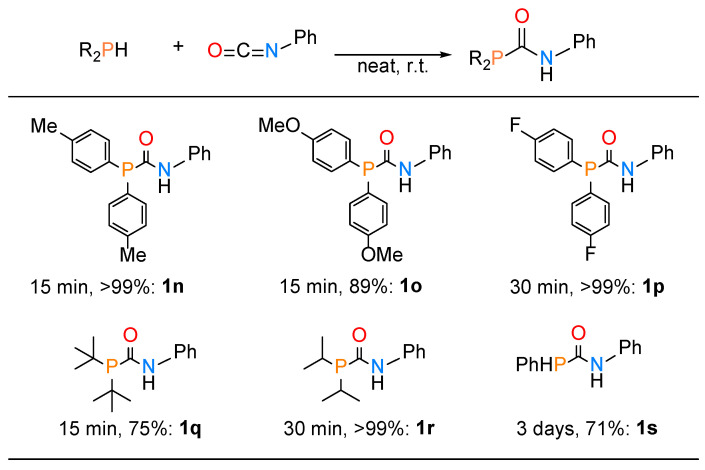
Hydrophosphination of phenylisocyanate with secondary phosphines R_2_PH and primary phosphine PhPH_2_ ^a,b^. ^a^ Phosphine compound: isocyanate = 1:1 molar ratio. ^b^ Isolated yield.

The results of the scope and limitation for the hydrophosphination of phenylisocyanate with phosphine compounds are summarized in Scheme 3. Diphenylphosphine analogs with electron-donating or -withdrawing groups such as Me, OMe, and F at the *para* position of the phenyl rings, and dialkylphosphines such as *^t^*Bu_2_PH and *^i^*Pr_2_PH were also adaptable to this reaction. In the case of primary phosphine PhPH_2_, single hydrophosphination product **1s** was obtained in 71%, but a long reaction time (3 days) was required because of the small amount of hexane existing in commercially available PhPH_2_ (see Section 3.1).

**Scheme 4:**
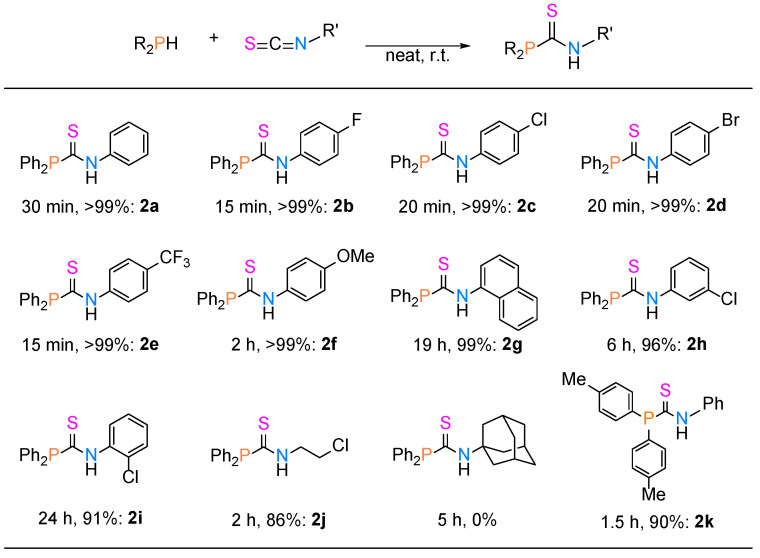
Scope and limitation for the hydrophosphination of isothiocyanates with diarylphosphine ^a,b^. ^a^ Phosphine compound: isothiocyanate = 1:1 molar ratio. ^b^ Isolated yield.

We reported the hydrophosphination of phenylisothiocyanate with diphenylphosphine to give the corresponding phosphinecarbothioamide **2a** in a previous study [27]. Very recently, the hydrophosphination of isothiocyanates using a catalyst-free method was reported by Zhu and Wang’s group [30]. For comparison, we also examined the synthesis of phosphinecarbothioamide without catalyst and solvent. Scheme 4 summarizes the results showing good functional group tolerance. Several isothiocyanates could be employed in the reaction, in which R is the phenyl ring with withdrawing groups such as F, Cl, Br, and CF_3_ at the *para* position (**2a**, **2b**, **2c**, **2d**, **2e**). These reactions were completed within 30 min. Using 4-methoxyphenylisothiocyanate, 1-naphthylisothiocyanate and 3-chlorophenylisocyanate, a long reaction time was required (**2f**, **2g**, **2h**). Surprisingly, the hydrophosphination reaction of 2-chlorophenylisothiocyanate took place. This result is in contrast with the hydrophosphination of 2-chlorophenylisocyanate. Isothiocyanate with an aliphatic substituent was also adaptable to this reaction (**2j**). 1-Adamathylisothiocyanate did not react. These tendencies are similar to those observed for the corresponding isocyanates except for 2-chlorophenylisocyanate. The hydrophosphination of phenylisothiocyanate with (4-methylphenyl)_2_PH also proceeded well (**2k**).

**Figure 2:**
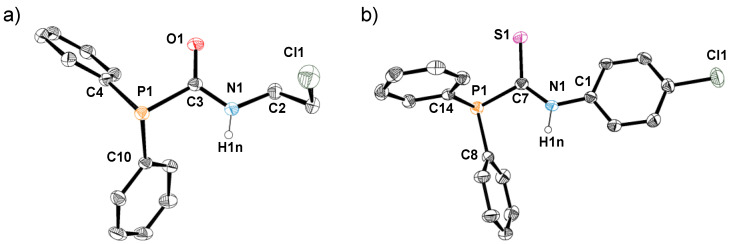
ORTEP drawings of (**a**) **1i** (P1 molecule) and (**b**) **2c** with thermal ellipsoids at 50% probability. Hydrogen atoms (except for NH protons) are omitted for simplicity. Selected bond lengths (Å) and angles (°): P1–C3 1.870(2), N1–C2 1.452(3), N1–C3 1.328(3), C3–O1 1.231(2), N1–H1n 0.81(3), C3–P1–C4 98.62(9), C3–P1–C10 103.32(9), C4–P1–C10 101.86(9), C2–N1–C3 123.30(19), C2–N1–H1n 121.1(19), C3–N1–H1n 115.4(19) for **1i** (P1 molecule); P1–C7 1.8541(17), N1–C1 1.420(2), N1–C7 1.336(2), C7–S1 1.6540(18), N1–H1n 0.86(2), C7–P1–C14 101.63(8), C7–P1–C8 102.88(7), C8–P1–C14 101.95(7), C1–N1–C7 132.20(16), C1–N1–H1n 110.5(13), C7–N1–H1n 117.3(13) for **2c**.

The molecular structures of **1i** and **2c** were determined using a single-crystal X-ray diffraction study. Two independent molecules of **1i** were crystallized in the unsymmetric unit. The ORTEP drawings are shown in Figure 2a) for **1i** (P1 molecule) and b) for **2c**, along with selected bond lengths and angles. The bond distances of P1–C3 (1.870(2) Å) and C3–N1 (1.328(3) Å) for **1i** were similar to those of P1–C7 (1.8541(17) Å) and N1–C7 (1.336(2) Å) for **2c**. The phosphinecarboxamide and phosphinecarbothioamide moieties displayed a pyramidalized geometry at the P atom (sum of angle around P1 = 330.8° for **1i** and 306.5° for **2c**), indicating that the π-electron conjugation of the C=O and C=S did not extend to the P. In contrast, the planar carboxamide and carbothioamide moieties (sum of angle around N1 = 359.9° for **1i** and 360.0° for **2c**) were observed, showing the delocalization of π-electron density between the carbonyl (thiocarbonyl) and amide groups. These were consistent with the previously reported phosphinecarboxamide [31].

**Scheme 5:**
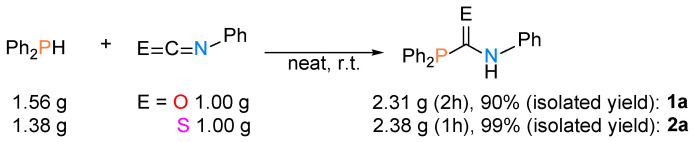
Gram-scale synthesis of phosphinecarboxamide and phosphinecarbothioamide.

It is noteworthy that gram-scale practical reactions were successfully performed: phosphinecarboxamide **1a** and phosphinecarbothioamide **2a** were isolated in 90% and 99% yields, respectively (Scheme 5).

### 2.2. Synthesis of Palladium(II) Complexes with Phosphinecarboxamide and Phosphinecarbothioamide

**Scheme 6:**
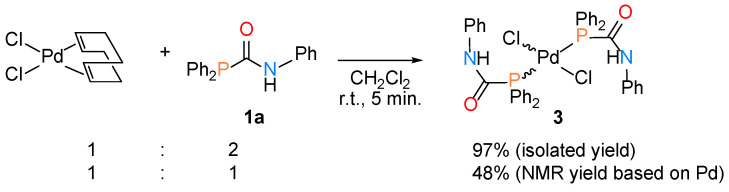
Reactions of Pd(COD)Cl_2_ with Ph_2_PC(O)NHPh **1a**.

The reaction of Pd(COD)Cl_2_ with **1a** at a 1:2 molar ratio produced the related bis(phosphinecarboxamide)palladium complex [Pd{Ph_2_PC(O)NHPh-κ*P*}_2_Cl_2_] (**3**) in 97% yield. Complex **3** was obtained even when Pd(COD)Cl_2_ was treated with 1 equiv. of **1a** (NMR yield: 48%) (Scheme 6). The NH proton signal of **1a** was not observed in CDCl_3_ because of the overlapped phenyl protons, but it was observed at δ 7.13 ppm in C_6_D_6_ [20]. In the ^1^H NMR spectrum of **3** in CDCl_3_, the NH proton was observed at δ 10.56 ppm. This chemical shift indicates that the NH of **3** is involved in hydrogen bonding. The ^31^P NMR spectrum of **3** showed two singlets (δ 25.5 ppm and 32.5 ppm in ca. 8:1 ratio) at 20 °C. The ratio of the two singlets depended on the temperature (ca. 16:1 at 50 °C and ca. 4:1 at −50 °C) (see Supplementary Materials, Figure S1). We think that this Pd complex adopts a planar geometry and performs *cis/trans* isomerization; the signal of δ 25.5 ppm was assigned to *trans-***3** and that of δ 32.5 ppm to *cis-***3**. The thermal *cis/trans* isomerization of bis(phosphine)palladium(II) complex is well known [32,33]. In our system, the thermodynamic parameters *ΔH*° (1.3 × 10^4^ Jmol^−1^) and *ΔS*° (6.2 × 10^1^ JK^−1^ mol^−1^) of **3** were obtained using van’t Hoff plots (see Supplementary Materials, Figure S2). These values were similar to those in the previous report [32,33].

**Figure 3:**
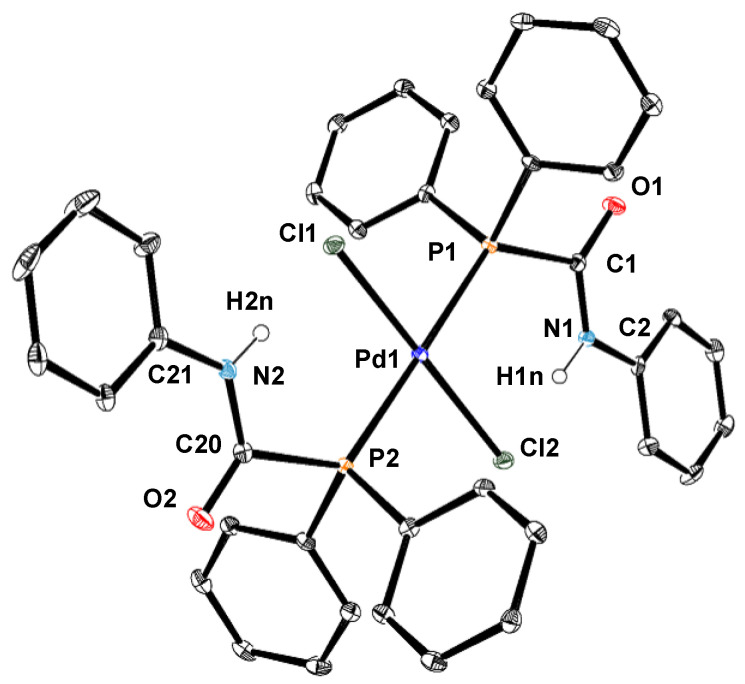
ORTEP drawing of *trans*-**3** with thermal ellipsoids at 30% probability. Hydrogen atoms (except for NH protons) are omitted for simplicity. Selected bond lengths (Å) and angles (°): Pd1–P1 2.3707(9), Pd1–P2 2.3574(9), Pd1–Cl1 2.3033(10), Pd1–Cl2 2.3132(10), C1–O1 1.218(4), C20–O2 1.218(4), N1–H1n 0.92(6), N2–H2n 0.83(4), P1–Pd1–P2 178.99(5), P1–Pd1–Cl1 82.33(4), P1–Pd1–Cl2 98.14(4), P2–Pd1–Cl1 96.70(4), P2–Pd1–Cl2 82.83(4), Cl1–Pd1–Cl2 179.29(4).

Since single crystals were obtained from the reaction solution of Scheme 6, an X-ray structure analysis was performed, and the ORTEP drawing was obtained, as depicted in Figure 3, with the atomic numbering scheme. The complex (*trans-***3**) was confirmed to adopt a typical square-planar configuration; the palladium center had two Cl atoms and two *P*-coordinated Ph_2_PC(O)NHPh (**1a**), which were *trans*-positioned with respect to each other. This molecule had two N–H···Cl intramolecular hydrogen bonds (Cl1···H2n (2.28(4) Å), Cl2···H1n (2.22(6) Å)). The hydrogen bonds in addition to the bulkiness of **1a** were considered to be the reason for the *trans* geometry. This is the first example of a planar complex in which two phosphinecarboxamide ligands are coordinated to the *trans* position. The Pd–P bond distances (2.3574(9), 2.3707(9) Å) were longer than those of previously reported for *cis*-[PdCl_2_{(±)-pbap}_2_] (pbap = 3-phenyl-1,3-dihydrobenzo [1,3]azaphosphol-2-one) (2.2420(7), 2.2282(7) Å) [2]. The sum of the angles around N1 and N2 atoms were ca. 360°. These angles were similar to those previously reported [31]. This observation indicates that the delocalization of π-electron density between the carbonyl and amide groups was maintained even after complex formation.

**Scheme 7:**
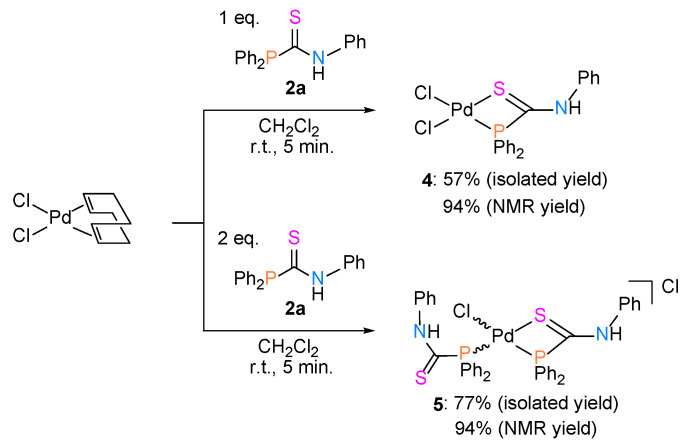
Reactions of Pd(COD)Cl_2_ with Ph_2_PC(S)NHPh **2a**.

**Figure 4:**
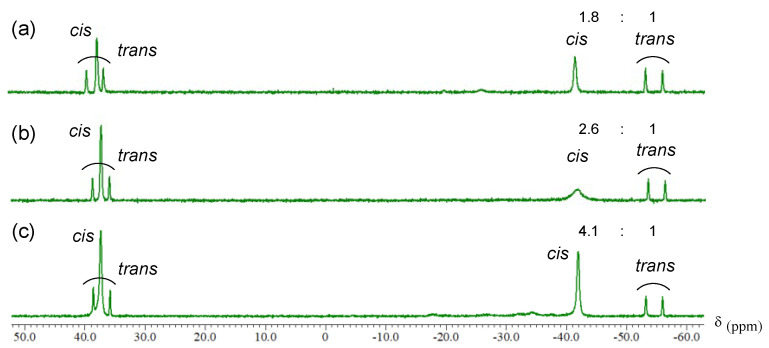
^31^P NMR spectra (162 MHz, CD_2_Cl_2_, ppm) of **5** at 20 °C (**a**), −10 °C (**b**), and −40 °C (**c**).

The reaction of Pd(COD)Cl_2_ with Ph_2_PC(S)NHPh (**2a**) at a 1:1 molar ratio produced [PdCl_2_{Ph_2_PC(S)NHPh-κ*P,S*}] (**4**) (57% yield) (Scheme 7 (upper)). In this complex, **2a** acted as a *P,S*-chelating bidentate ligand. The ^31^P{^1^H} NMR spectrum of **4** showed a singlet at δ –41.3 ppm. On the other hand, the reaction of Pd(COD)Cl_2_ with **2a** at a 1:2 molar ratio produced [PdCl{Ph_2_PC(S)NHPh-κ*P,S*}{Ph_2_PC(S)NHPh-κ*P*}]Cl (**5**) in 77% yield (Scheme 7 (lower)). Although the NH proton signal of **2a** was observed at δ 8.64 ppm in CDCl_3_, the corresponding signals of **4** and **5** in CDCl_3_ were observed at δ 13.31 ppm and 13.00 ppm, respectively. These large downfield shifts of the NH proton show the hydrogen bonding between the NH proton and the Cl atom in solution. The ^31^P NMR spectra of **5** at various temperatures (20, −10, and −40 °C) are displayed in Figure 4. Coupled two doublets with ^2^*J*(PP) = 453.4 Hz were observed at δ –54.2 ppm, 37.8 ppm and those with ^2^*J*(PP) = 26.2 Hz were observed at δ –41.9 ppm, 37.3 ppm at 20 °C. We assigned the signals with the largest coupling constant to the *trans* complex and those with the smallest coupling constant to the *cis* complex. The ratio of the *cis* complex increased when the measurement temperature was lowered (*ca.* 1.8:1 at 20 °C, 2.6:1 at −10 °C, and ca. 4.1:1 at −40 °C), and this ratio changed reversibly with the temperature. This behavior has not been reported in previous complexes having phosphinecarbothioamide. Thermodynamic parameters *ΔH*° (8.6 × 10^3^ Jmol^−1^) and *ΔS*° (2.5 × 10^1^ JK^−1^ mol^−1^) of **5** were obtained using van’t Hoff plots and were similar to the values found for **3** (see Supplementary Materials, Figure S3).

**Figure 5:**
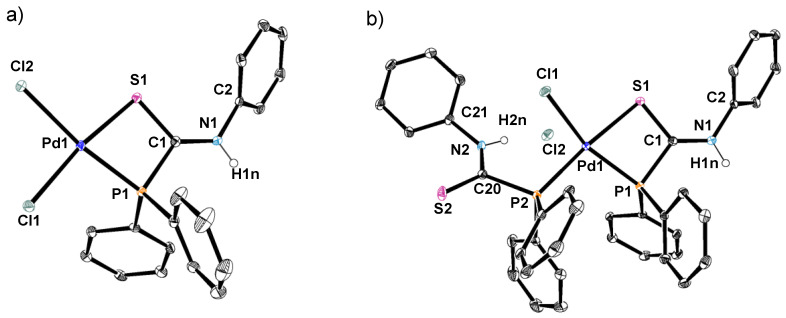
ORTEP drawings of (**a**) **4**·0.5(CH_3_)_2_CO and (**b**) *cis-***5**·CH_2_Cl_2_ with thermal ellipsoids at 30% probability. The crystal solvent and hydrogen atoms (except for NH protons) are omitted for simplicity. Selected bond lengths (Å) and angles (°): Pd1–P1 2.2064(7), Pd1–S1 2.2954(8), Pd1–Cl1 2.3299(7), Pd1–Cl2 2.3534(7), S1–C1 1.704(2), C1–N1 1.302(3), C1–P1 1.844(2), N1–H1n 0.83(2), P1–Pd1–S1 75.47(3), P1–Pd1–Cl1 96.05(3), P1–Pd1–Cl2 171.75(2), S1–Pd1–Cl1 171.48(2), S1–Pd1–Cl2 96.35(3), Cl1–Pd1–Cl2 92.14(3) for **4**; Pd1–P1 2.2324(7), Pd1–P2 2.2886(7), Pd1–S1 2.3448(7), Pd1–Cl1 2.3688(6), S1–C1 1.693(2), C1–N1 1.305(3), C1–P1 1.838(2), N1–H1n 0.89(3), P2–C20 1.876(2), C20–S2 1.651(2), C20–N2 1.324(3), N1–H1n 0.84(3), P1–Pd1–P2 100.41(3), P1–Pd1–S1 74.66(2), P1–Pd1–Cl1 159.01(2), P2–Pd1–S1 174.45(2), P1–C1–S1 103.63(11) for **5**.

**Figure 6:**
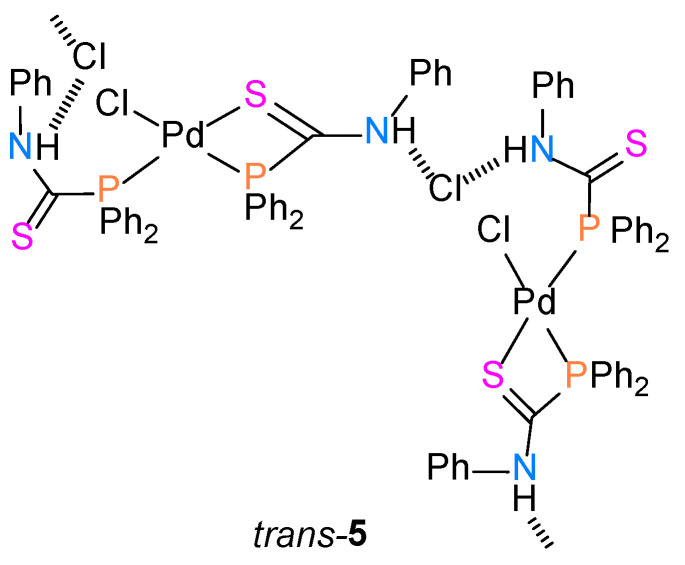
Inter-molecular hydrogen bonds (hash line) for *trans-***5**.

The structures of **4** and **5** were studied via an X-ray crystal structure analysis. Two independent molecules of **4** crystallized in the unit cell. As these were basically the same, only one molecule (Pd1) is shown in Figure 5a). Complex **4** had a typical square-planar environment; palladium had two Cl atoms situated mutually in *cis* position and *P,S-*coordinated bidentate ligand **2a**. The bond lengths of Pd–P (2.2064(7), 2.1887(7) Å), Pd–S (2.2954(8), 2.2928(8) Å), and Pd–Cl (2.3299(7), 2.3986(7) Å) were similar to those of the previously reported [PdCl_2_{Ph_2_PC(S)NMe_2_-κ*P,S*}] complex (2.209(1) Å for Pd–P bond, 2.290(1) Å for Pd–S bond, and 2.329(1), 2.376(1) Å for Pd–Cl bond) [11]. Intermolecular hydrogen bonds between the H1n atoms for the Pd1 molecule and the Cl4 atom for the Pd2 molecule were observed (H1n···Cl4 (2.28(2) Å)). Compound **5** consisted of a cationic palladium complex and a Cl^–^ counter anion (Figure 5b)). In the cationic Pd complex, the Pd atom is coordinated to the phosphorus atom of **2a** as a monodentate ligand, a bidentate *P,S-*bonded **2a**, and a Cl atom, acquiring a square-planar configuration. The two phosphorus atoms were *cis* with respect to each other. This is the first example of a transition-metal complex with both monodentate and bidentate phosphinecarbothioamide ligands. Complex **5** also showed inter-molecular hydrogen bonds (2.18(3) Å for H1n···Cl2 and 2.29(3) Å for H2n···Cl2) (Figure 6) [25].

## 3. Materials and Methods

### 3.1. General Considerations

The synthesis of phosphinecarboxamide and phosphinecarbothioamide was carried out using standard Schlenk techniques in a dry nitrogen atmosphere. The synthesis of complexes **3**–**5** was carried out in air. Iron complex CpFe(CO)_2_(Me) [34] and palladium complex PdCl_2_(COD) [35] were prepared according to the literature method. The other chemicals were commercially available. Phenylphosphine PhPH_2_ (10% hexane solution) was used after distillation (however, a little amount of hexane could not be removed). Solvents were purified employing a two-column solid-state purification system or were distilled from appropriate drying agents under N_2_. Spectroscopic data of the products obtained in this work, **1a**–**1g**, **2a**–**2d, 2f**, and **2k**, agreed with those in the literatures [16,17]. Spectroscopic data of the products **1i**–**1s** were preliminarily reported in [27]. NMR spectra (^1^H, ^13^C, and ^31^P) were recorded at ambient temperature with a JNM ECS-400 spectrometer. ^1^H and ^13^C NMR data were referred to residual peaks of solvent as an internal standard. Peak positions of ^31^P NMR spectra were referenced to external 85% H_3_ PO_4_ (δ = 140 ppm). Elemental analysis data were obtained with a Perkin–Elmer 2400 CHN elemental analyzer.

### 3.2. Synthesis

#### 3.2.1. General Procedure for Synthesis of Phosphinecarboxamide and Phosphinecarbothioamide

The mixture of the phosphine compound (0.7 mmol) and isocyanate/isothiocyanate (0.7 mmol) was stirred at room temperature in a Schlenk tube. After the reaction was complete, all volatile materials were removed under reduced pressure. The residual powder was dried in vacuo to give the corresponding phosphinecarboxamide/phosphinecarbothioamide.

*m*-ClC_6_H_4_NHC(O)PPh_2_ (**1h**): Ph_2_PH (0.7 mmol, 122 μL) was treated with 3-chlorophenylisocyanate (0.7 mmol, 85 μL) using a general procedure to give the product **1h** (236 mg, 99%) as a white powder. ^1^H NMR (400 MHz, CDCl_3_, ppm) δ 7.06–7.07 (m, 1H), 7.16–7.21 (m, 1H), 7.30 (br, 1H), 7.45–7.46 (m, 7H), 7.51–7.53 (m, 1H), 7.57–7.61 (m, 4H). ^13^C NMR (100.4 MHz, CDCl_3_, ppm) δ 117.6, 119.7, 124.9, 129.3 (d, ^3^*J*_C-P_ = 7.47 Hz), 130.1, 130.4, 132.8 (d, ^1^*J*_C-P_ = 10.8 Hz), 134.5 (d, ^2^*J*_C-P_ = 19.1 Hz), 134.8, 138.6, 176.6 (d, ^1^*J*_C-P_ = 16.6 Hz, C=O). ^31^P{^1^H} NMR (161.70 MHz, CDCl_3_, ppm) δ –0.76 (s). Elemental analysis (%) calcd for C_19_H_15_ClNOP: C, 67.17; H, 4.45; N, 4.12. Found: C, 67.20; H, 4.58; N, 3.96%.

*p*-CF_3_C_6_H_4_NHC(S)PPh_2_ (**2e**): Ph_2_PH (0.7 mmol, 122 μL) was treated with 4-trifluoromethylphenylisothiocyanate (0.7 mmol, 142 mg) using a general procedure to give the product **2e** (270 mg, 99%) as a yellow powder. ^1^H NMR (400 MHz, CDCl_3_, ppm) δ 7.45–7.51 (m, 6H), 7.55–7.61 (m, 6H), 7.80 (d, 2H, ^3^*J*_H-H_ = 8.8 Hz), 8.78 (br, 1H, NH). ^13^C NMR (100.4 MHz, CDCl_3_, ppm) δ 122.1, 126.3 (q, *J* = 3.8 Hz), 129.6 (d, ^3^*J*_C-P_ = 6.7 Hz), 130.7, 134.3, 134.5, 134.7, 141.6, 208.5 (d, ^1^*J*_C-P_ = 40.3 Hz, C=S). The CF_3_ peak overlapped with another peak. ^19^F NMR (376.95 MHz, CDCl_3_, ppm) δ 9.20 (s). ^31^P{^1^H} NMR (161.70 MHz, CDCl_3_, ppm) δ 20.5 (s). Elemental analysis (%) calcd for C_20_H_15_F_3_NSP: C, 61.69; H, 3.88; N, 3.60. Found: C, 61.74; H, 4.00; N, 3.57%.

*m*-ClC_6_H_4_NHC(S)PPh_2_ (**2h**): Ph_2_PH (0.7 mmol, 122 μL) was treated with 3-chlorophenylisothiocyanate (0.7 mmol, 92 μL) using a general procedure to give the product **2h** (240 mg, 96%) as a yellow powder. ^1^H NMR (400 MHz, CDCl_3_, ppm) δ 7.20–7.22 (m, 1H), 7.26–7.30 (m, 1H), 7.45–7.50 (m, 7H), 7.54–7.58 (m, 4H), 7.74–7.75 (m, 1H), 8.66 (br, 1H, NH). ^13^C NMR (100.4 MHz, CDCl_3_, ppm) δ 120.6, 122.4, 127.2, 129.5 (d, ^3^*J*_C-P_ = 7.67 Hz), 130.1, 130.6, 134.3, 134.5, 134.5 (d, ^2^*J*_C-P_ = 20.3 Hz), 139.8, 207.9 (d, ^1^*J*_C-P_ = 40.3 Hz, C= S) ppm. ^31^P{^1^H} NMR (161.70 MHz, CDCl_3_, ppm) δ 19.4 (s). Elemental analysis (%) calcd for C_19_H_15_ClNSP: C, 64.14; H, 4.25; N, 3.94 found: C, 63.92; H, 4.33; N, 3.86%.

*o*-ClC_6_H_4_NHC(S)PPh_2_ (**2i**): The mixture of diphenylphosphine Ph_2_PH (0.7 mmol, 122 μL) and 2-chlorophenylisothiocyanate (0.7 mmol, 92 μL) was stirred at room temperature in a Schlenk tube. After 24 h, all volatile materials were removed under reduced pressure. The residue was washed with *n*-hexane (1 mL × 3) at −70 °C and dried in vacuo to give **2i** (226 mg, 91%) as a yellow powder. ^1^H NMR (400 MHz, CDCl_3_, ppm) δ 7.16 (t, 1H, ^3^*J*_H-H_ = 7.8 Hz), 7.28–7.35 (m, 2H), 7.45–7.46 (m, 6H), 7.59–7.62 (m, 4H), 8.95 (d, 1H, ^3^*J*_H-H_ = 7.8 Hz), 9.15 (br, 1H, NH). ^13^C NMR (100.4 MHz, CDCl_3_, ppm) δ 123.6, 125.8, 127.2, 127.6, 129.5 (d, ^3^*J*_C-P_ = 7.47 Hz), 129.7, 130.6, 134.4 (d, ^1^*J*_C-P_ = 15.8 Hz), 134.8 (d, ^2^*J*_C-P_ = 20.8 Hz), 135.6, 208.2 (d, ^1^*J*_C-P_ = 39.9 Hz, C=S). ^31^P{^1^H} NMR (161.70 MHz, CDCl_3_, ppm) δ 22.2 (s). Elemental analysis (%) calcd for C_19_H_15_ClNSP: C, 64.14; H, 4.25; N, 3.94. Found: C, 63.97; H, 4.32; N, 3.78%.

ClC_2_H_4_NHC(S)PPh_2_ (**2j**): The mixture of Ph_2_PH (0.7 mmol, 122 μL) and 2-chloroethylisothiocyanate (0.7 mmol, 68 μL) was stirred at room temperature in a Schlenk tube. After 2 h, all volatile materials were removed under reduced pressure. The residual yellow powder was washed with *n*-hexane (1 mL × 3) at –70 °C and dried in *vacuo* to give **2j** (185 mg, 86%). ^1^H NMR (400 MHz, CDCl_3_, ppm) δ 3.53 (t, 2H, *J* = 9.2 Hz, CH_2_), 4.58–4.63 (m, 2H, CH_2_), 7.46–7.55 (m, 6H), 7.58–7.62 (m, 4H). The peak of NH overlapped with another peak. ^13^C NMR (100.4 MHz, CDCl_3_, ppm) δ 32.2, 58.0, 129.6 (d, ^3^*J*_C-P_ = 8.3 Hz, *m*-Ph), 131.7, 134.6, 134.9, 202.2 (d, ^1^*J*_C-P_ = 59.0 Hz, C=S). ^31^P{^1^H} NMR (161.70 MHz, CDCl_3_, ppm) δ –4.20 (s). Elemental analysis (%) calcd for C_15_H_15_ClNSP: C, 58.54; H, 4.91; N, 4.55. Found: C, 57.99; H, 4.97; N, 4.44%.

#### 3.2.2. NMR Tube Experiments for Pd(II) Complexes

A CDCl_3_ solution (0.5 mL) of Pd(COD)Cl_2_ (5.8 mg, 0.020 mmol), Ph_2_PC(O)NHPh **1a** (6.2 mg, 0.020 mmol) and P(=O)Ph_3_ (internal standard, 5.3 mg, 0.019 mmol) was mixed in an NMR tube at room temperature. After 5 minutes, ^31^P NMR was measured, which revealed that [Pd{Ph_2_PC(O)NHPh-κ*P*}_2_Cl_2_] (**3**) was formed in 48% NMR yield based on Pd.

A CDCl_3_ solution (0.5 mL) of Pd(COD)Cl_2_ (7.1 mg, 0.025 mmol), Ph_2_PC(S)NHPh **2a** (8.1 mg, 0.025 mmol) and P(=O)Ph_3_ (internal standard, 8.1 mg, 0.029 mmol) was mixed in an NMR tube at room temperature. After 5 minutes, ^31^P NMR was measured, which revealed that [PdCl_2_{Ph_2_PC(S)NHPh-κ*P,S*}] (**4**) was formed in 94% NMR yield.

A CDCl_3_ solution (0.5 mL) of Pd(COD)Cl_2_ (7.1 mg, 0.025 mmol), Ph_2_PC(S)NHPh **2a** (16.1 mg, 0.050 mmol), and P(=O)Ph_3_ (internal standard, 8.1 mg, 0.029 mmol) was mixed in an NMR tube at room temperature. After 5 minutes, ^31^P NMR was measured, which revealed that [PdCl{Ph_2_PC(S)NHPh-κ*P,S*}{(*P*-)Ph_2_PC(S)NHPh}]Cl (**5**) was formed in 94% NMR yield.

#### 3.2.3. Synthesis of Pd(II) Complexes

[Pd{Ph_2_PC(O)NHPh-κ*P*}_2_Cl_2_] (**3**): A dichloromethane solution (2.5 mL) in a flask containing PdCl_2_(COD) (57.2 mg, 0.20 mmol) and Ph_2_PC(O)NHPh **1a** (122.3 mg, 0.40 mmol) was stirred at room temperature. After 5 min, all volatile materials were removed under reduced pressure. The residual yellow powder was washed with *n*-hexane and dried in vacuo to give **3** (153.7 mg, 0.195 mmol, 97%) as a yellow powder. ^1^H NMR (400 MHz, CDCl_3_, ppm) δ 7.11–7.16 (m, 2H), 7.28–7.30 (m, 4H), 7.46–7.50 (m, 8H), 7.54–7.59 (m, 8H), 7.81–7.86 (m, 8H), 10.56 (br, 2H, NH). ^13^C{^1^H} NMR (100.4 MHz, CDCl_3_, ppm) δ 120.3 (s), 125.6 (s), 126.3 (vt, *J*_C-P_ = 24.9 Hz), 128.7 (vt, *J* = 5.3 Hz), 129.2 (s), 131.8 (s), 135.0 (vt, *J* = 6.2 Hz), 137.6 (vt, *J* = 4.8 Hz), 164.9 (vt, *J* = 26.4 Hz, C=O). ^31^P{^1^H} NMR (162 MHz, CDCl_3_, ppm) δ 25.5 (s). Elemental analysis (%) calcd for C_38_H_32_Cl_2_N_2_O_2_P_2_Pd: C, 57.92; H, 4.09; N, 3.56. Found: C, 57.66; H, 4.22; N, 3.50%.

[PdCl_2_{Ph_2_PC(S)NHPh-κ*P,S*}] (**4**): A dichloromethane solution (1.0 mL) in a test tube containing PdCl_2_(COD) (14.3 mg, 0.050 mmol) and Ph_2_PC(S)NHPh **2a** (32.2 mg, 0.100 mmol) was stirred at room temperature for 5 min. Yellow crystal **4** (14.3 mg, 0.029 mmol, 57%) was obtained by solvent diffusion over a few days from a CH_2_Cl_2_ layer and an overlayer of hexane. ^1^H NMR (400 MHz, −50 °C, CDCl_3_, ppm) δ 7.36–7.53 (m, 10H), 7.88–7.90 (m, 2H), 7.99–8.04 (m, 3H), 13.31 (brs, NH, 1H). ^13^C{^1^H} NMR (100.4 MHz, 20 °C, CDCl_3_, ppm) δ 119.8 (s), 120.3 (s), 123.7 (s), 129.6 (s), 129.9 (d, *J*_C-P_ = 12.5 Hz), 134.0 (s), 135.0 (d, *J*_C-P_ = 13.4 Hz), 135.5 (d, *J*_C-P_ = 4.8 Hz), The C=S peak was not observed. ^31^P{^1^H} NMR (162 MHz, 20 °C, CDCl_3_, ppm) δ −41.3 (s). Elemental analysis (%) calcd for C_19_H_16_Cl_2_NPPdS: C, 45.76; H, 3.23; N, 2.81. Found: C, 45.97; H, 3.57; N, 2.76%.

[PdCl{Ph_2_PC(S)NHPh-κ*P,S*}{(*P*-)Ph_2_PC(S)NHPh}]Cl (**5**). A dichloromethane solution (1.0 mL) in a test tube containing PdCl_2_(COD) (14.3 mg, 0.050 mmol) and Ph_2_PC(S)NHPh **2a** (32.2 mg, 0.10 mmol) was stirred at room temperature for 5 min. The orange powder **5** (35.0 mg, 0.039 mmol, 77%) was obtained via solvent diffusion over a few days from a CH_2_Cl_2_ layer and an overlayer of hexane. ^1^H NMR (400 MHz, –40 °C, CD_2_Cl_2_, ppm) δ 6.08 (brs, NH, 1H), 7.14–7.18 (m, 8H), 7.35–7.43 (m, 19H), 8.09–8.11 (m, 3H), 13.0 (brs, NH, 1H). ^31^P{^1^H} NMR (162 MHz, –40 °C, CD_2_Cl_2_, ppm) δ 37.8 (d, *J* = 453.4 Hz, *trans*), 37.3 (d, *J* = 26.2 Hz, *cis*), –41.9 (d, *J* = 26.2 Hz, *cis*), –54.2 (d, *J* = 453.4 Hz, *trans*). Elemental analysis (%) calcd for C_38_H_32_Cl_2_N_2_P_2_PdS_2_: C, 55.66; H, 3.93; N, 3.42. Found: C, 55.99; H, 4.30; N, 3.33%.

### 3.3. Crystallography

Crystallographic data are summarized in Table 1. The single crystals of **1i**, **2c**, and **3**–**5** were obtained using the slow diffusion method (CH_2_Cl_2_/hexane for **3** and **5**; acetone/ether for **4**). Diffraction-intensity data were collected with Rigaku AFC11 with a Saturn 724 + CCD diffractometer (200(2) K for **1i**, **2c**; 110(2) K for **3**–**5**), and semiempirical multi-scan absorption correction [36] was performed. The structures were solved using SIR97 [37] via subsequent difference Fourier synthesis, and refined with full matrix least-squares procedures on F^2^. All non-hydrogen atoms were refined with anisotropic displacement coefficients. NH protons were determined via difference Fourier synthesis and refined isotropically. Hydrogen atoms (except for NH protons) were treated as idealized contributions and refined in a rigid group model. All software and sources of scattering factors were contained in the SHELXL-2018/3 [38] program package. The Cambridge Crystallographic Data Centre (CCDC) deposition numbers of **1i**, **2c**, and **3**–**5** are included in Table 1.

## 4. Conclusions

We achieved the catalyst-free synthesis of phosphinecarboxamide and phosphinecarbothioamide through the hydrophosphination of isocyanates and isothiocyanates. The important point in our system was to carry out the reaction without using a solvent (neat). This system showed the characteristics of easy handling, high yield, short reaction time, and good functional-group tolerance for the functionalized isocyanates and isothiocyanates. For the requirement of no catalyst nor solvent, the gram-scale synthesis of these compounds was also achieved. In addition, we synthesized and characterized palladium complexes with phosphinecarboxamide and phosphinecarbothioamide as ligands. In the reaction of Pd(COD)Cl_2_ with **1**a, [Pd{Ph_2_PC(O)NHPh-κ*P*}_2_Cl_2_] (**3**) was obtained regardless of whether **1a** was used in 1 or 2 equivalents. In contrast, in the reaction of Pd(COD)Cl_2_ with **2a**, [PdCl_2_{Ph_2_PC(S)NHPh-κ*P,S*}] (**4**) and [PdCl{Ph_2_PC(S)NHPh-κ*P,S*}{Ph_2_PC(S)NHPh-κ*P*}]Cl (**5**) were selectively obtained when 1 eq. and 2 eq. of **2a** were used, respectively. It was revealed that **3** and **5** having intra- and/or inter-molecular hydrogen bonds showed thermal *cis/trans* isomerization.

## Figures and Tables

**Table 1:** Crystallographic data and details of structure refinement parameters of **1i**, **2c**, and **3**–**5**

	1i	2c	3	4·0.5(CH_3_)_2_CO	5·CH_2_Cl_2_
empirical formula	C_15_H_15_ClNOP	C_19_H_15_ClNPS	C_38_H_32_Cl_2_N_2_O_2_P_2_Pd	C_20.5_H_19_Cl_2_NO_0.5_PSPd	C_39_H_34_Cl_4_N_2_P_2_S_2_Pd
formula weight	291.70	355.80	787.89	527.70	904.94
*T* (K)	200(2)	200(2)	110(2)	110(2)	110(2)
crystal system	triclinic	monoclinic	orthorhombic	triclinic	monoclinic
space group	$P\bar{1}$	*P*2_1_*/n*	*Pca*2_1_	$P\bar{1}$	*P*2_1_/*n*
*a* (Å)	9.29470(10)	9.2578(5)	12.9390(2)	9.679(3)	17.288(5)
*b* (Å)	11.8190(2)	21.2798(9)	12.4521(3)	14.927(4)	13.508(3)
*c* (Å)	21.0466(4)	9.9399(5)	21.0466(4)	15.962(4)	18.189(5)
*α* (°)	85.569(6)			106.543(4)	
*β* (°)	82.125(7)	116.3332(15)		92.7607(7)	112.506(3)
*γ* (°)	67.021(6)			103.748(3)	
volume (Å^3^)	1448.16(9)	1755.00(15)	3390.98(12)	2130.6(10)	3924.0(17)
Z	4	4	4	4	4
*ρ*_calcd_ (mg m^–3^)	1.338	1.347	1.543	1.645	1.532
*μ* (mm^–1^)	0.365	0.426	0.837	1.303	0.965
*F*(000)	608	736	1600	1056	1832
crystal size (mm^3^)	0.37 × 0.27 × 0.12	0.27 × 0.18 × 0.17	0.12 × 0.07 × 0.02	0.13 × 0.07 × 0.03	0.21 × 0.16 × 0.06
reflections collected	17,154	13,335	27,555	22,263	39,512
R(int)	6564 (0.0361)	3974 (0.0253)	5973 (0.0309)	9665 (0.0288)	8955 (0.0336)
*R*1 (*I* > 2σ(*I*))	0.0478	0.0503	0.0204	0.0278	0.0346
w*R*2 (all data)	0.1187	0.1062	0.0502	0.0600	0.0704
goodness of fit	1.106	1.094	1.042	1.046	1.094
CCDC deposition number	1959302	2193371	2168755	2168782	2168783

## Data Availability

The crystallographic data are available from the Cambridge Crystallographic Data Centre (CCDC). Other data not presented in Supplementary Materials are available upon request from the corresponding author.

## Supplementary Materials

The following supporting information can be downloaded at: https://www.mdpi.com/article/10.3390/molecules27175564/s1, Figure S1: ^31^P NMR spectra (CDCl_3_, 161.70 MHz) of **3** at 50 °C, 20 °C, and −50 °C, Figure S2: van’t Hoff plots for **3**, Figure S3: van’t Hoff plots for **5**, Figures S4–S27: NMR spectra of all new compounds.

## Sample Availability

Samples of the compounds are not available from the authors.
