# Supplementary material for: Synthesis and Characterization of Phosphinecarboxamide and Phosphinecarbothioamide, and Their Complexation with Palladium(II) Complex
Source: Molecules. 2022 Aug 29;27(17):5564. doi: 10.3390/molecules27175564 (PMC9457633; doi:10.3390/molecules27175564)
Supplement: Supplementary file 1 [file molecules-27-05564-s001.zip › molecules-1871017-supplementary.pdf]

SUPPORTING INFORMATION

Table of Contents **Page**

^{31}P NMR spectra ($CDCl_3$, 161.70 MHz) of **3** at 50 °C, 20 °C, and−50 °C S2

van't Hoff plots for **3** and **5** S2

NMR spectra of all new compounds S3-S9

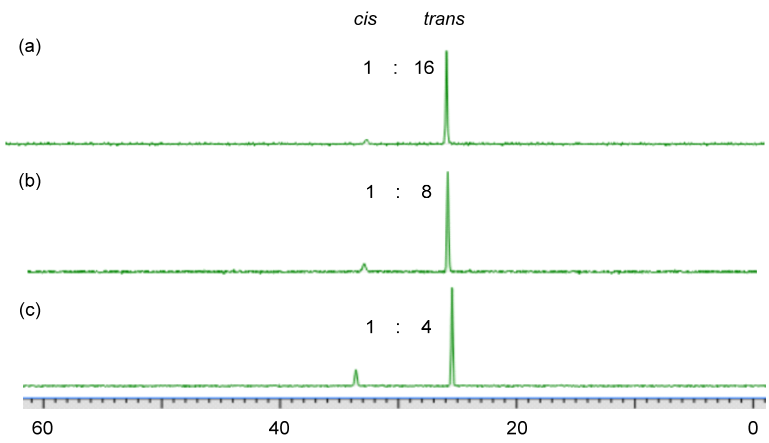

Figure S1. ^{31}P NMR spectra (161.70 MHz, $CDCl_3$) of **3** at 50 °C (a), 20 °C (b), and−50 °C (c).

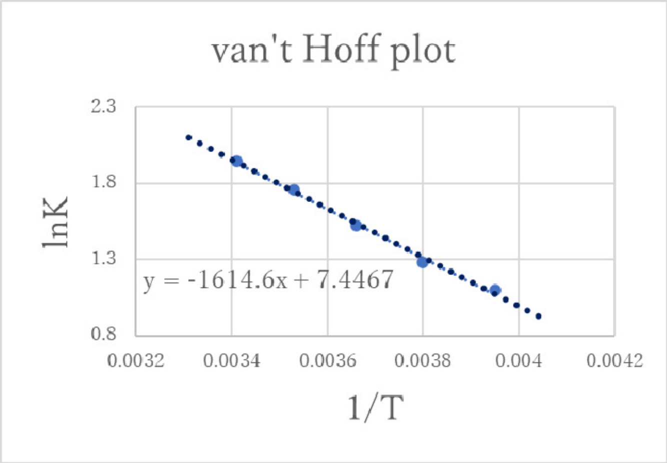

Figure S2. van’t Hoff plots for **3**.

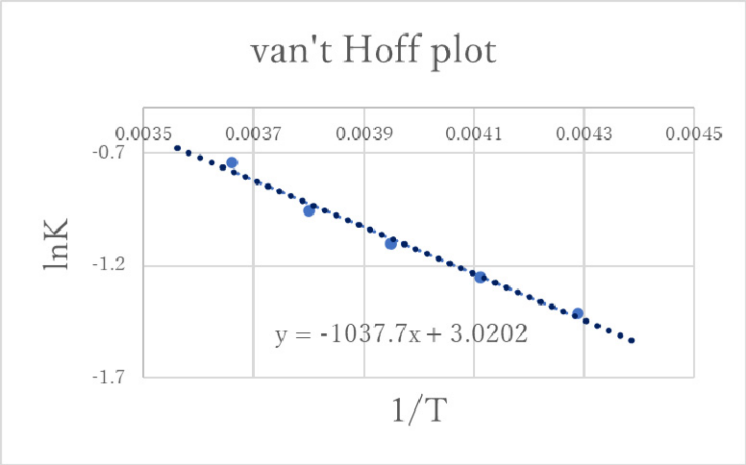

Figure S3. van’t Hoff plots for **5**.

NMR spectra of all new compounds

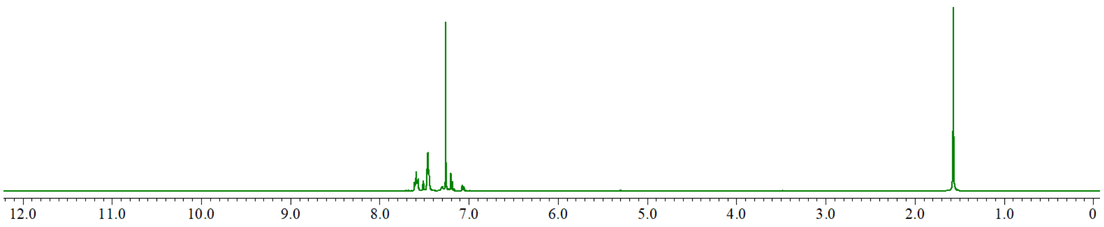

Figure S4. ^{1}H NMR spectrum (400 MHz, $CDCl_3$, 20 °C) of **1h**.

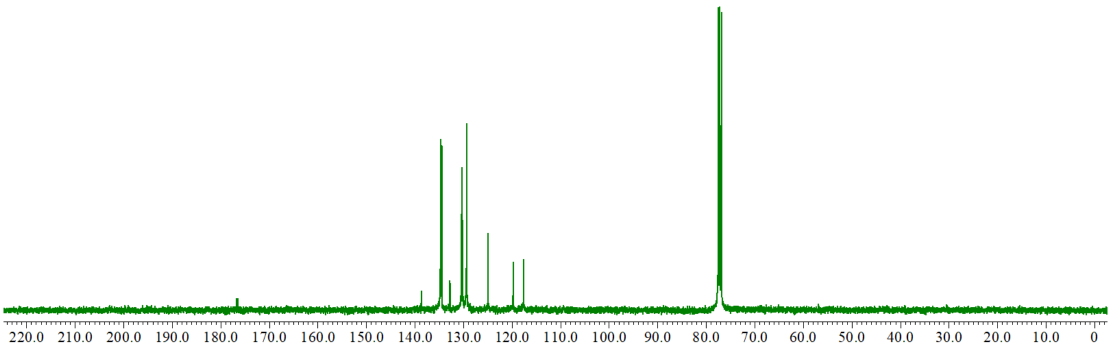

Figure S5.^{13}C NMR spectrum (100.4 MHz, $CDCl_3$, 20 °C) of **1h**.

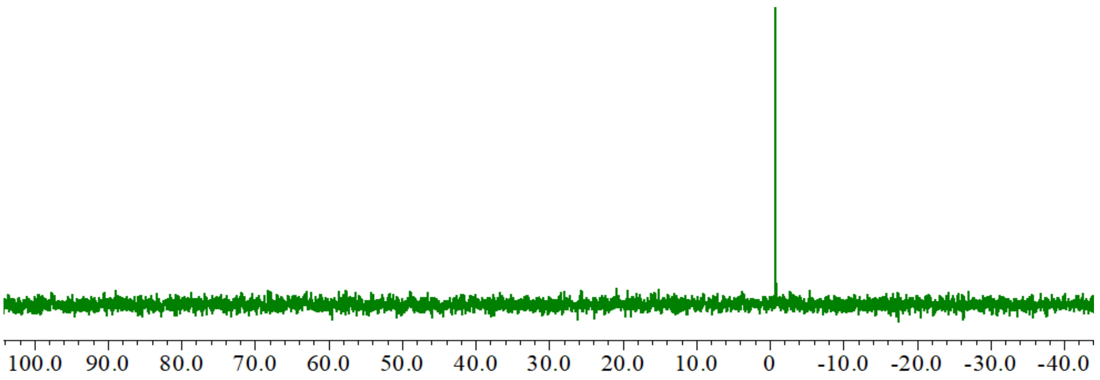

Figure S6. $^{31}P\{^{1}H\}$ NMR spectrum (161.70 MHz, $CDCl_3$, 20 °C) of **1h**.

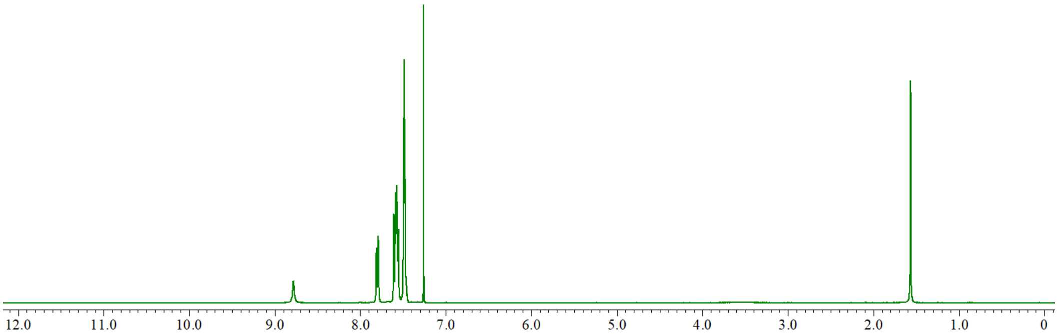

Figure S7. ^{1}H NMR spectrum (400 MHz, $CDCl_3$, 20 °C) of **2e**.

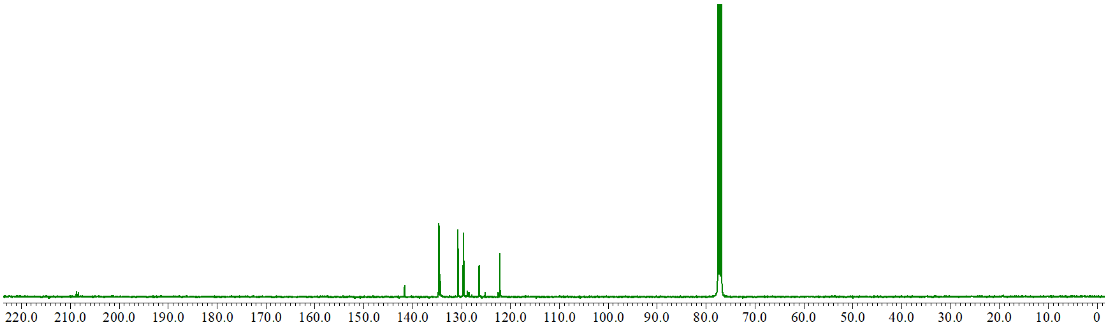

Figure S8. ^{13}C NMR spectrum (100.4 MHz, $CDCl_3$, 20 °C) of **2e**.

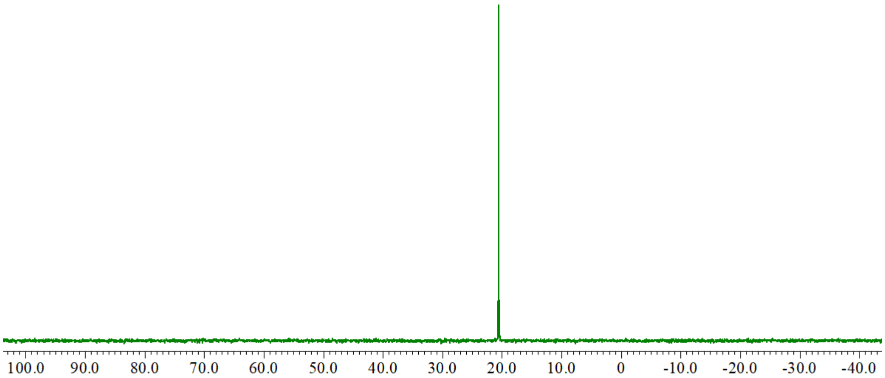

Figure S9. $^{31}P\{^{1}H\}$ NMR spectrum (161.70 MHz, $CDCl_3$, 20 °C) of **2e**.

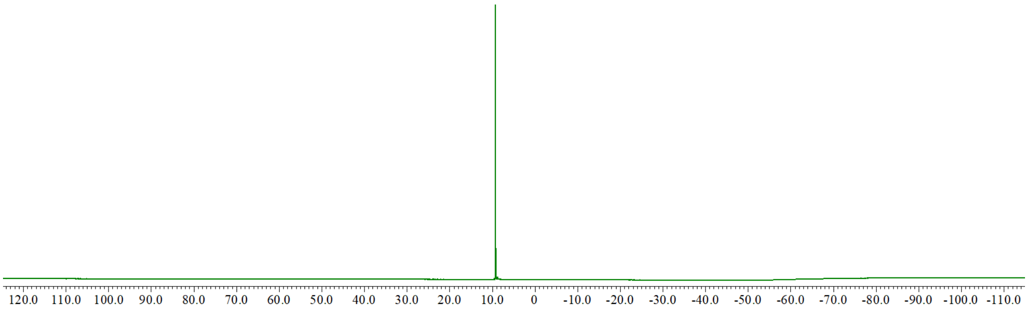

Figure S10. ^{19}F NMR spectrum (376.95 MHz, $CDCl_3$, 20 °C) of **2e**.

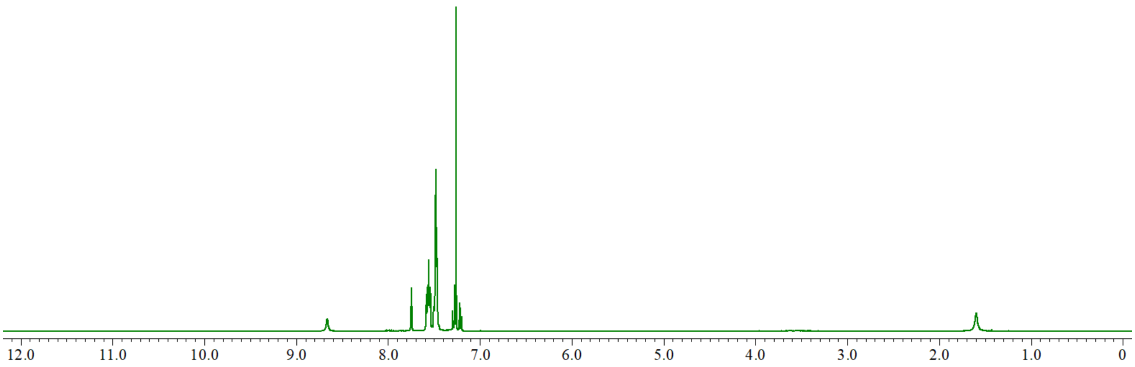

Figure S11. ^{1}H NMR spectrum (400 MHz, $CDCl_3$, 20 °C) of **2h**.

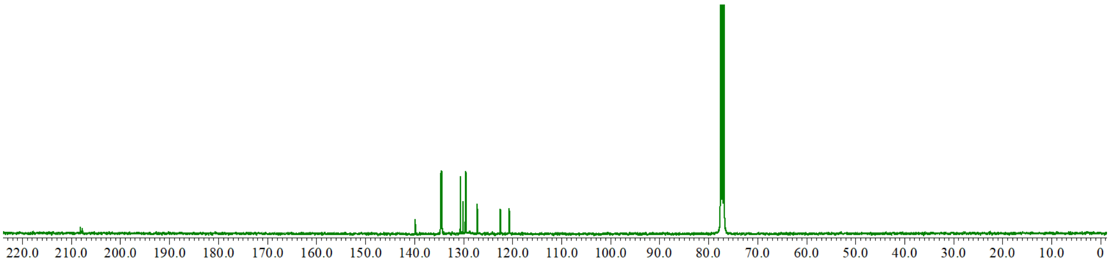

Figure S12. ^{13}C NMR spectrum (100.4 MHz, $CDCl_3$, 20 °C) of **2h**.

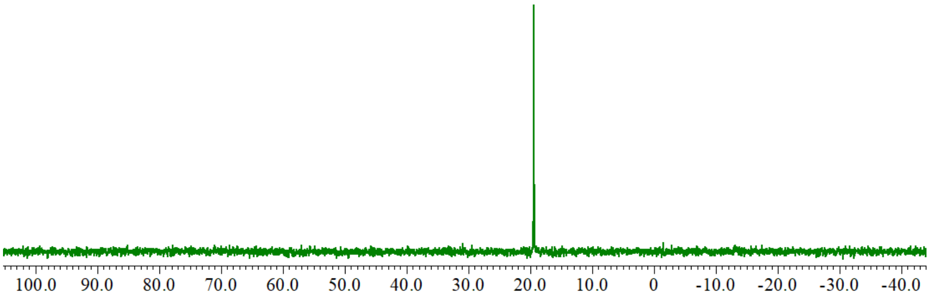

Figure S13. $^{31}P\{^{1}H\}$ NMR spectrum (161.70 MHz, $CDCl_3$, 20 °C) of **2h**.

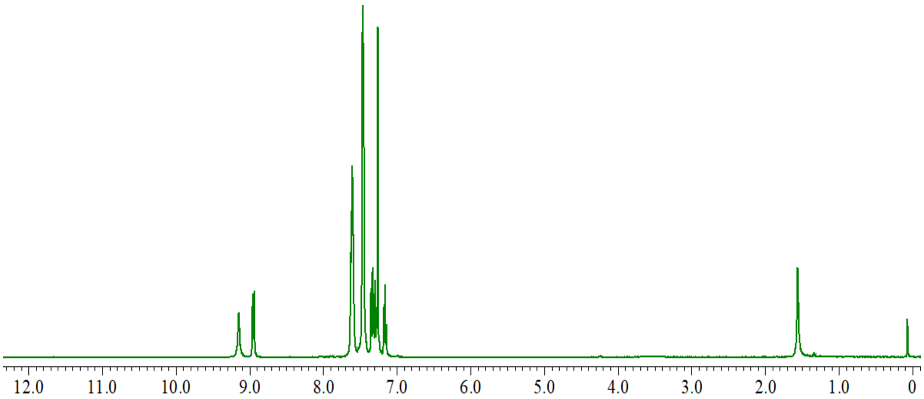

Figure S14. ^{1}H NMR spectrum (400 MHz, $CDCl_3$, 20 °C) of **2i**.

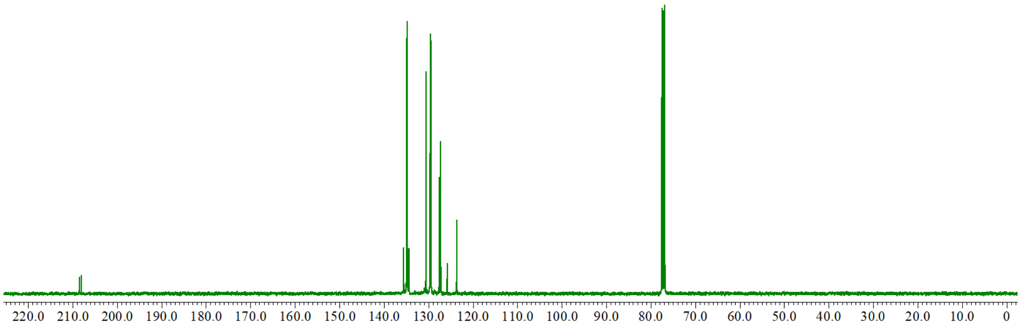

Figure S15. ^{13}C NMR spectrum (100.4 MHz, $CDCl_3$, 20 °C) of **2i**.

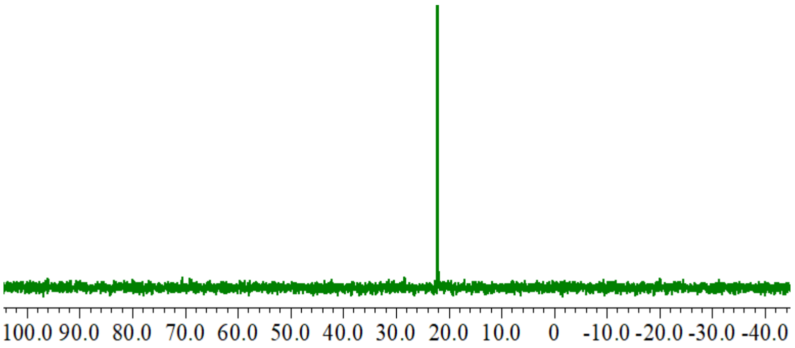

Figure S16. $^{31}P\{^{1}H\}$ NMR spectrum (161.70 MHz, $CDCl_3$, 20 °C) of **2i**.

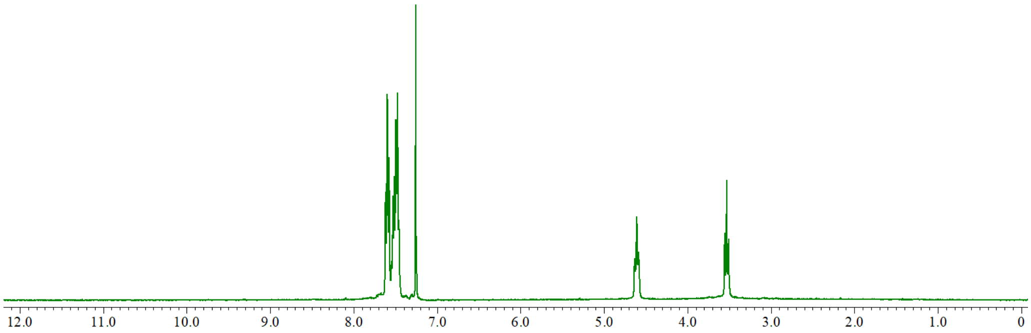

Figure S17. ^{1}H NMR spectrum (400 MHz, $CDCl_3$, 20 °C) of **2j**.

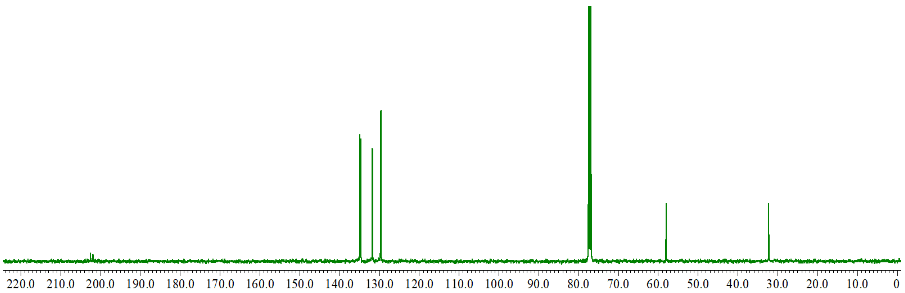

Figure S18. ^{13}C NMR spectrum (100.4 MHz, $CDCl_3$, 20 °C) of **2j**.

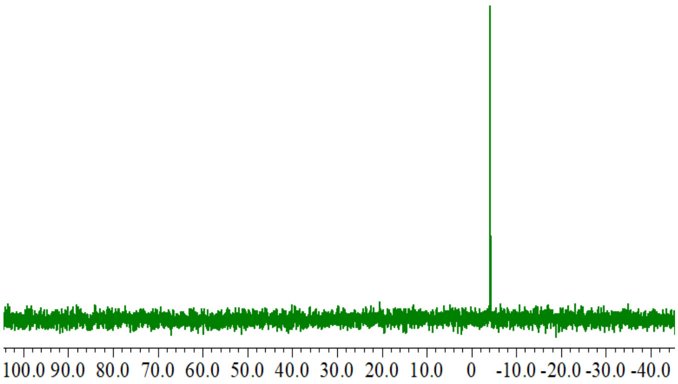

Figure S19. $^{31}P\{^{1}H\}$ NMR spectrum (161.70 MHz, $CDCl_3$, 20 °C) of **2j**.

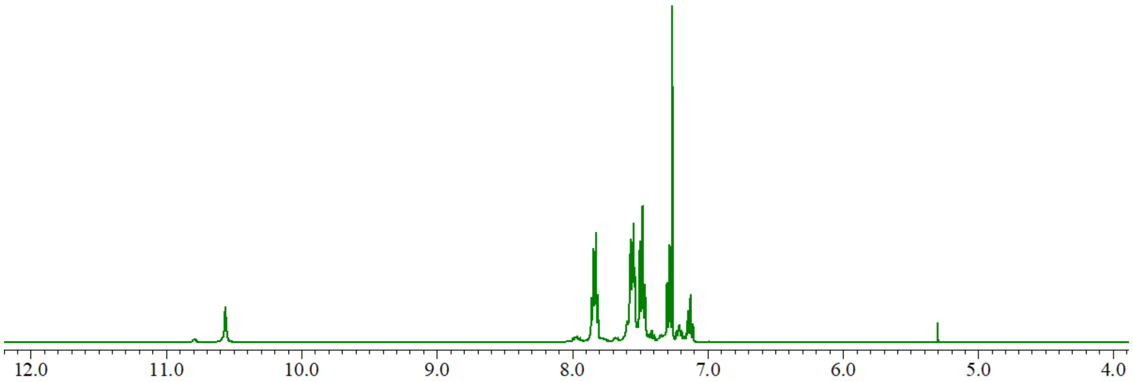

Figure S20. ^{1}H NMR spectrum (400 MHz, $CDCl_3$, 20 °C) of **3**.

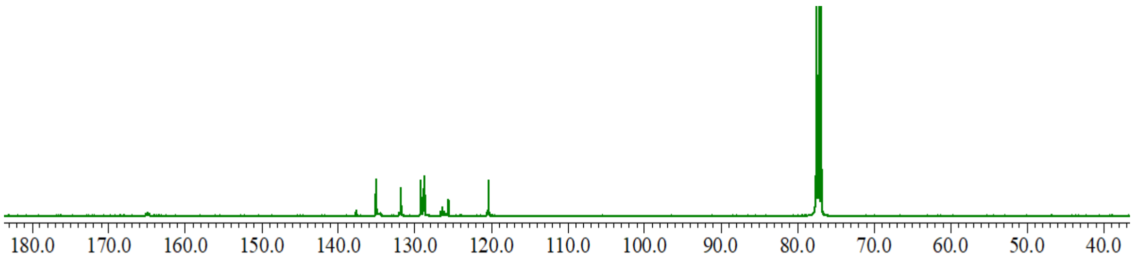

Figure S21. $^{13}C\{^{1}H\}$ NMR spectrum (100.4 MHz, $CDCl_3$, 20 °C) of **3**.

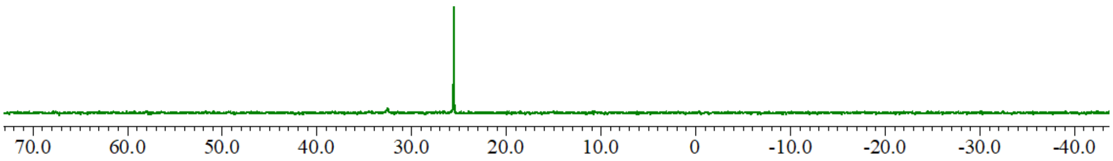

Figure S22. $^{31}P\{^{1}H\}$ NMR spectrum (162 MHz, $CDCl_3$, 20 °C) of **3**.

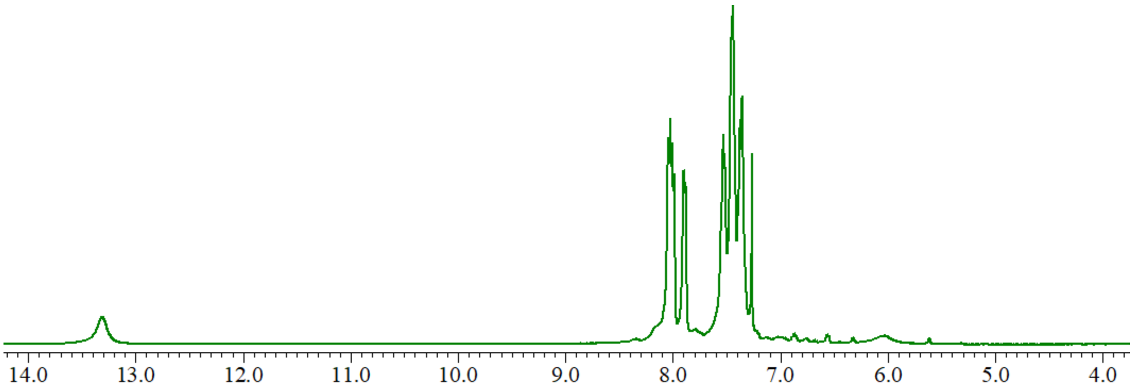

Figure S23. ^{1}H NMR spectrum (400 MHz, $CDCl_3$, –50 °C) of **4**.

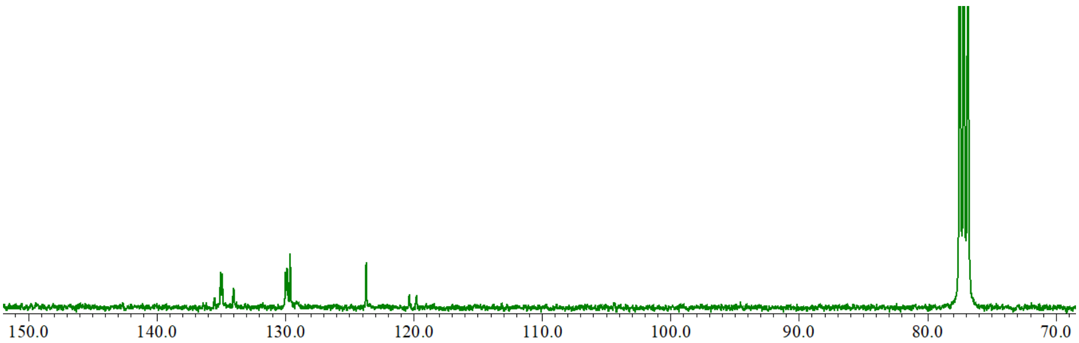

Figure S24. $^{13}C\{^{1}H\}$ NMR spectrum (100.4 MHz, $CDCl_3$, 20 °C) of **4**.

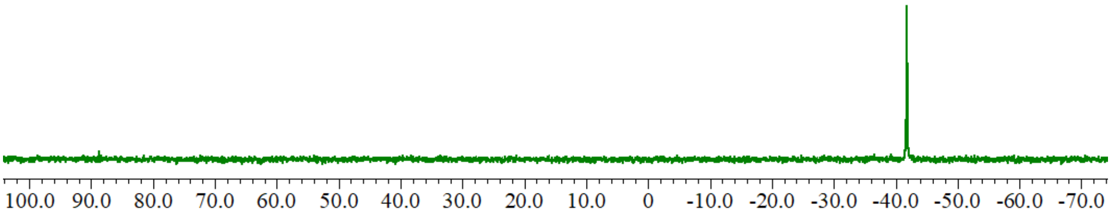

Figure S25. $^{31}P\{^{1}H\}$ NMR spectrum (162 MHz, $CDCl_3$, 20 °C) of **4**.

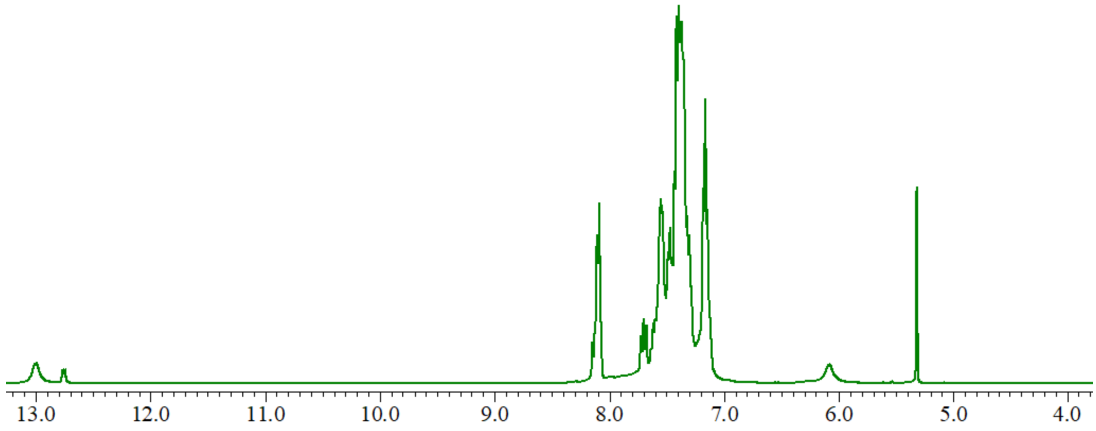

Figure S26. ^{1}H NMR spectrum (400 MHz, CD_2Cl_2, –40 °C) of **5**.

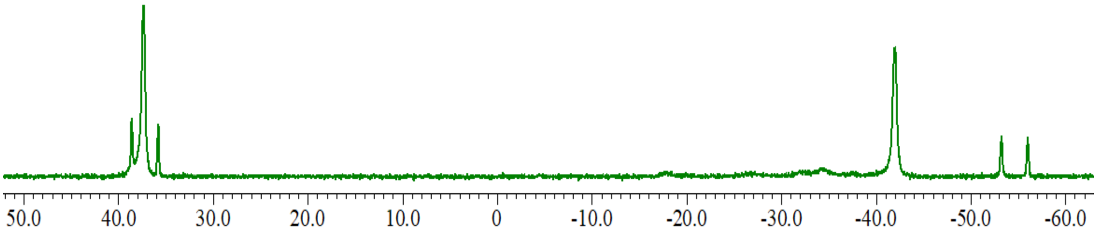

Figure S27. $^{31}P\{^{1}H\}$ NMR spectrum (162 MHz, CD_2Cl_2, –40 °C) of **5**.
